# The distinctive structure and composition of arterial and venous thrombi and pulmonary emboli

**DOI:** 10.1038/s41598-020-59526-x

**Published:** 2020-03-20

**Authors:** Irina N. Chernysh, Chandrasekaran Nagaswami, Sofia Kosolapova, Alina D. Peshkova, Adam Cuker, Douglas B. Cines, Carolyn L. Cambor, Rustem I. Litvinov, John W. Weisel

**Affiliations:** 1 University of Pennsylvania School of Medicine, Philadelphia, PA USA; 2 Kazan Federal University, Kazan, Russian Federation

**Keywords:** Embolism, Thrombosis

## Abstract

Although arterial and venous thromboembolic disorders are among the most frequent causes of mortality and morbidity, there has been little description of how the composition of thrombi and emboli depends on their vascular origin and age. We quantified the structure and composition of arterial and venous thrombi and pulmonary emboli using high-resolution scanning electron microscopy. Arterial thrombi contained a surprisingly large amount of fibrin, in addition to platelets. The composition of pulmonary emboli mirrored the most distal part of venous thrombi from which they originated, which differed from the structure of the body and head of the same thrombi. All thrombi and emboli contained few biconcave red blood cells but many polyhedrocytes or related forms of compressed red blood cells, demonstrating that these structures are a signature of clot contraction *in vivo*. Polyhedrocytes and intermediate forms comprised the major constituents of venous thrombi and pulmonary emboli. The structures within all of the thrombi and emboli were very tightly packed, in contrast to clots formed *in vitro*. There are distinctive, reproducible differences among arterial and venous thrombi and emboli related to their origin, destination and duration, which may have clinical implications for the understanding and treatment of thrombotic disorders.

## Introduction

Thromboembolism remains a leading cause of death and disability. Thrombi may partially or totally obstruct arteries or veins, leading to local ischemic complications and they can embolize to the cerebral arteries and lungs, where they may cause stroke or other life-threatening conditions. There are approximately 1 million incident cases of deep venous thromboembolism each year and over 60,000 deaths per year from recognized pulmonary embolism in the United States alone^1,2^. Over 900,000 patients are hospitalized each year for management of acute myocardial infarction, which is responsible for 1 in every 6 deaths in the US, and about 800,000 patients suffer from stroke each year with 87% of all strokes being ischemic. Stroke kills about 140,000 people each year, or 1 in 20 deaths in the US^3^. Insights into the pathophysiology of thrombotic disorders have come from studies using a variety of designs, ranging from detailed *in vitro* molecular and cellular approaches, *ex vivo* pathological studies, *in silico* multiscale thrombosis modeling to *in vivo* animal models and clinical trials. Yet, there are remarkably few detailed analyses of thrombus structure that further our understanding of their relationship to vascular origin and duration *in vivo*, as well as the composition or the internal structural features of thrombi most closely associated with the risk of embolization.

The composition, physical properties, and evolution of venous and arterial thrombi are likely to differ mainly due to various local conditions and time since formation. Platelets play an important role in the development of arterial thrombi formed at relatively high wall shear rates (~10^2^–10^5^ s^−1^), generating what are often termed “white” thrombi. In patients with coronary artery disease, thrombi commonly arise from the rupture of atherosclerotic plaque and exposure of procoagulant components, such as collagen and lipid-rich activated macrophages bearing tissue factor^4^, leading to myocardial infarction if thrombi become obstructive. Similar events may lead to *in situ* arterial thrombosis in the cerebral or other circulations. In contrast, venous thrombi formed under low shear rate (10–100 s^−1^) are mainly composed of red blood cells (RBCs) and fibrin, i.e. “red” thrombi. The formation of venous thrombi is generally attributed to a combination of hypercoagulability together with injured or activated endothelium and impaired blood flow (Virchow’s triad)^5,6^. Whether these perceived differences in pathogenesis affect thrombus structure has received relatively little investigation but may be important for the risk of extension and embolization and approach to therapy.

Thrombi also undergo structural changes as they age. For example, the composition of coronary artery thrombi obtained from patients with ST-elevation myocardial infarction evolve over time such that fibrin content doubles each hour during clinically manifesting ischemia, whereas the relative platelet content halves each hour^7,8^. These changes have been associated with formation of a dense, stiff fibrin network that impairs responsiveness to anti-platelet therapy and thrombolysis over time^9,10^. A similar increase in stiffness over time associated with changes in structure occurs with aging of venous thrombi^11^. Knowing more about changes in thrombus structure over time is desirable, because they could influence the treatment approach and prognosis.

Until recently, RBCs have been viewed as passive bystanders in these processes. It is generally thought that RBCs are trapped in venous clots and might thereby increase vascular occlusion, but that they otherwise contribute little to arterial occlusion. There is now increasing evidence that RBCs play a substantial role in clotting^12^. Sub-fractions of RBCs express phosphatidylserine on their surface and thus support thrombin generation^13,14^. RBCs also suppress plasmin generation and as a result inhibit clot lysis^15,16^. Platelet-driven clot contraction results in compression of RBCs into a tightly packed interior core with an accompanying shape change to polyhedral cells, named polyhedrocytes, whereas fibrin and platelets are mostly on the surface^17^. Polyhedrocytes have also been observed in intracoronary thrombi taken from patients post ST-elevation myocardial infarction^8,17,18^ as well as in *ex vivo* thrombi, venous clots and postmortem pulmonary emboli^12,19,20^. *In vitro* studies of clot contraction have demonstrated that the kinetics of clot contraction are accelerated and extent of contraction is enhanced by higher platelet levels and inhibited by RBCs and higher fibrinogen concentrations^21^.

Despite the clinical importance, a comprehensive analysis of these and the above-mentioned features to human arterial and venous thrombi and emboli has not been reported.

In this study, we used high resolution scanning electron microscopy to examine how the vascular origin and aging affect the structure and composition of *in vivo* human arterial and venous thrombi extracted from living patients and postmortem pulmonary emboli. We determined the proportions composed of different forms of fibrin, the presence of biconcave, polyhedral and intermediate compressed forms of RBCs, and echinocytes, as well as volume fractions of the thrombi comprised by leukocytes, platelets, and cellular microvesicles. These results revealed unique characteristics and differences between arterial and venous thrombi as well as between their younger and older thrombi. These differences in composition among various types of thrombi not only reflect the mechanisms of their formation but also have implications for why some thrombi embolize and differ in their responsiveness to antithrombotic treatments.

## Results

### Approach

We used high resolution scanning electron microscopy to examine the structure and composition of the exterior and interior of 45 freshly aspirated arterial thrombi, 25 venous thrombi from open thrombectomy, and 10 postmortem pulmonary emboli (for technical details of obtaining thrombi and patient characteristics see the Supplemental Material and Methods). We imaged 10–12 randomly selected areas and collected 10–12 images from each specimen, resulting in about 700 total images. We studied these images without any pre-determined criteria to develop a set of “typical structural elements” found in any or all types of thrombi and emboli. We then selected representative images from randomly chosen portions of 6 arterial thrombi, 5 venous thrombi, and 6 pulmonary emboli that contained all the typical structural elements seen in all the specimens, and the overall composition and structural elements contained in these portions were quantified. The following structural elements were included in the analysis: fibrin (individual fibers, bundles, sponge); individual platelets, platelet aggregates and degranulated platelets; RBCs (biconcave, polyhedral, intermediate forms, balloon-like forms, echinocytes); white blood cells (WBCs); cellular microvesicles; and space between structures. In sum, total image areas of 29,600 µm^2^ from arterial thrombi, 55,100 µm^2^ from venous thrombi, and 38,600 µm^2^ from pulmonary emboli were quantified (Supplemental Fig. S1).

### Differential composition of arterial and venous thrombi and pulmonary emboli

#### Composition of arterial thrombi

**Figure 1:**
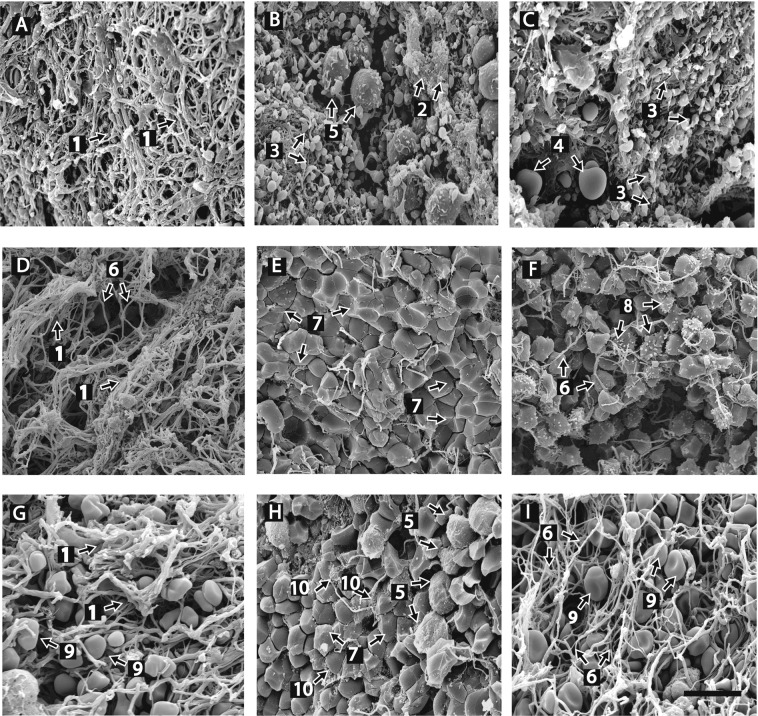
Representative scanning electron microscope images of thrombi and emboli. Structures identified in arterial and venous thrombi and pulmonary emboli. Panels A–C are images of arterial thrombi. Panels D–F are images of venous thrombi. Panels G–I are images of pulmonary emboli. (**A**) Arterial thrombus: fibrin structure is primarily composed of fiber bundles (1) and (**B**) fibrin sponge (2). (**B**,**C**) Dense contracted thrombi with platelet aggregates (3) with fibrin on the outside and red blood cell balloons (4) trapped in the fibrin mesh; some white blood cells were also present (5). (**D**) Venous thrombus, mostly fibrin structure is primarily composed of fiber bundles (1) and individual fibrin fibers (6). (**E**) Tightly packed RBCs in the form of polyhedrocytes (7) with a few fibrin fibers. (**F**) Fibrin fibers (6); RBCs present as polyhedrocytes and echinocytes (8). (**G**) Pulmonary embolus, bundles of fibrin (1) with intermediate forms of RBCs trapped inside (9). (**H**) Mostly polyhedrocytes (7) and extracellular microvesicles (10) as well as many white blood cells are present (5). (**I**) Individual fibrin fibers (6) and intermediate forms of RBCs (9). Magnification bar = 10 µm.

**Figure 2:**
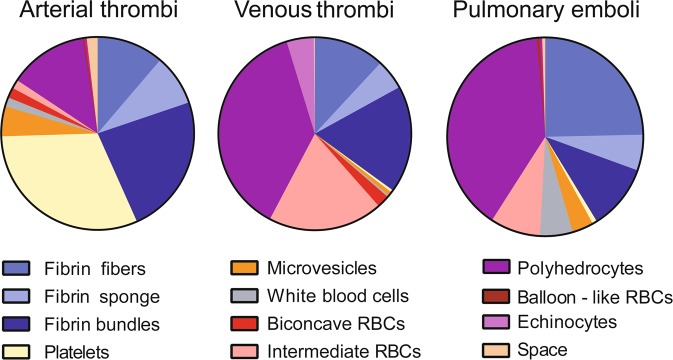
Quantitation of structures identified in pulmonary emboli, arterial and venous thrombi. Thrombi were obtained from patients and prepared for scanning electron microscopy and their composition was quantified as described in the Materials and Methods section. The fibrin component was present as individual fibrin fibers, fibrin sponge and fibrin bundles; erythrocytes as biconcave, intermediate shapes, polyhedrocytes, echinocytes and balloon-like forms. The proportions of total volume occupied by these structures were calculated and presented as pie charts. RBC = red blood cell.

Coronary artery thrombi were composed primarily of fibrin (43% of the thrombus volume) and platelets (31%) (Figs. 1A–C and 2, Table 1). However, the appearance of the fibrin was surprisingly inhomogeneous and differed considerably from clots formed *in vitro*, which are generally composed of a branching network of individual thin fibers^22^. In the arterial thrombi, fibrin was found predominantly in the form of bundles (55% of all fibrin types), some of which were unusually thick. In addition, there was also a highly branched network of very thin fibers that we have termed “fibrin sponge”. These arterial thrombi were dense, meaning little unoccupied space was present. They also showed evidence of clot contraction, in that polyhedrocytes were commonly interspersed among fiber bundles^17^. During clot contraction, platelets pull on fibrin fibers and compress RBCs, resulting in a transformation of shape from biconcave cells to polyhedral and various intermediate forms with formation of fiber bundles^17^. Other RBCs appear balloon-shaped, as if they had been squeezed through the network of fibrin fibers or were otherwise deformed, as reported previously^23,24^. Of the 17% of the volume composed of RBCs, most (15%) comprised polyhedrocytes or other compressed forms, with smaller proportions of normal biconcave RBCs (2%) and balloon-like RBCs (less than 1%). Cellular microvesicles and leukocytes occupied 5% and less than 2% of the thrombus volume, respectively.

**Table 1:** The average volume fraction (%) of structures identified in arterial and venous thrombi and pulmonary emboli.

Structures	Arterial Thrombi	Venous Thrombi	Pulmonary Emboli
Total Fibrin	43.3	35.0	41.2
Single Fibers	11.2	12.0	24.7
Bundles	23.6	17.9	10.7
Sponge	8.5	5.1	5.8
Platelets	31.3	0.4	0.8
Microvesicles	5.0	0.7	3.5
White Blood Cells	1.5	0.3	5.4
Total RBC	17.1	63.4	48.6
Biconcave	1.7	2.1	0.0
Echinocytes	0.4	4.6	0.4
Compressed	15.0	56.7	48.2
Polyhedrocytes	13.2	37.5	39.4
Intermediate	1.5	19.2	8.3
Balloon-like	0.3	0.0	0.5
Space	1.8	0.2	0.5

#### Composition of venous thrombi

The overall composition of venous thrombi differed significantly from arterial thrombi. RBCs and fibrin fibers were the major components of venous thrombi, comprising on average 63% and 35% of the volume, respectively, while arterial thrombi were mostly composed of fibrin and platelets (Table 1; Fig. 1G–I). RBC content was significantly higher in venous thrombi than in arterial thrombi (p < 0.0001). The proportion of compressed RBCs was also higher in venous thrombi than in arterial thrombi (p < 0.0001). Most of the RBC component was present as polyhedrocytes and intermediate-shaped cells (38% and 19% of all RBCs, respectively), while the corresponding percentage in arterial thrombi was 13% and 2%. Only 2% of the cells in venous thrombi were biconcave discs. Surprisingly, 5% of venous thrombus volume was occupied by echinocytes, which are characterized by spike-like or thorny projections. Such changes could be caused by long-term metabolic changes or alteration of their environment after thrombus formation. The fibrin was heterogeneous in composition, with 12%, 5% and 18% in the form of single fibrin fibers, fibrin sponge, and fibrin bundles, respectively. The platelet content (0.4%) was significantly lower than in arterial thrombi (31.3%, p < 0.0001). The remainder of the volume was made up of microvesicles (1%) and leukocytes (less than 1%) (Table 1; Fig. 2).

#### Composition of pulmonary emboli

The Chi-square test showed that overall composition of pulmonary emboli differed significantly from arterial thrombi (p < 0.0001) but was not significantly different from venous thrombi (p = 0.061), although some individual differences were significant. The proportions of volume occupied by RBCs and platelets were especially different from the respective volumes they occupied in arterial thrombi (p < 0.0001 for both). There were slight differences of emboli with parental venous thrombi, namely they were both composed primarily of fibrin fibers and RBCs. However, the volumes occupied by these structures in the emboli (41% and 49% of the overall composition, respectively) were different from the venous thrombi (Table 1; Fig. 2). Sixty percent of the total fibrin component in emboli was composed of individual fibrin fibers, which is significantly more than in venous thrombi (p = 0.0025). Overall, the RBC content was lower in pulmonary emboli than in venous thrombi, 63% versus 49% (p = 0.046). Another notable difference was that polyhedrocytes comprised a larger portion of RBC population (81%) compared with venous thrombi (59%) (p = 0.0053). The volumes occupied by platelets (0.8%), microvesicles (4%), and leukocytes (5%) were similar to the proportions in venous thrombi (Table 1; Fig. 2).

### Individual components

#### Fibrin content and structure

**Figure 3:**
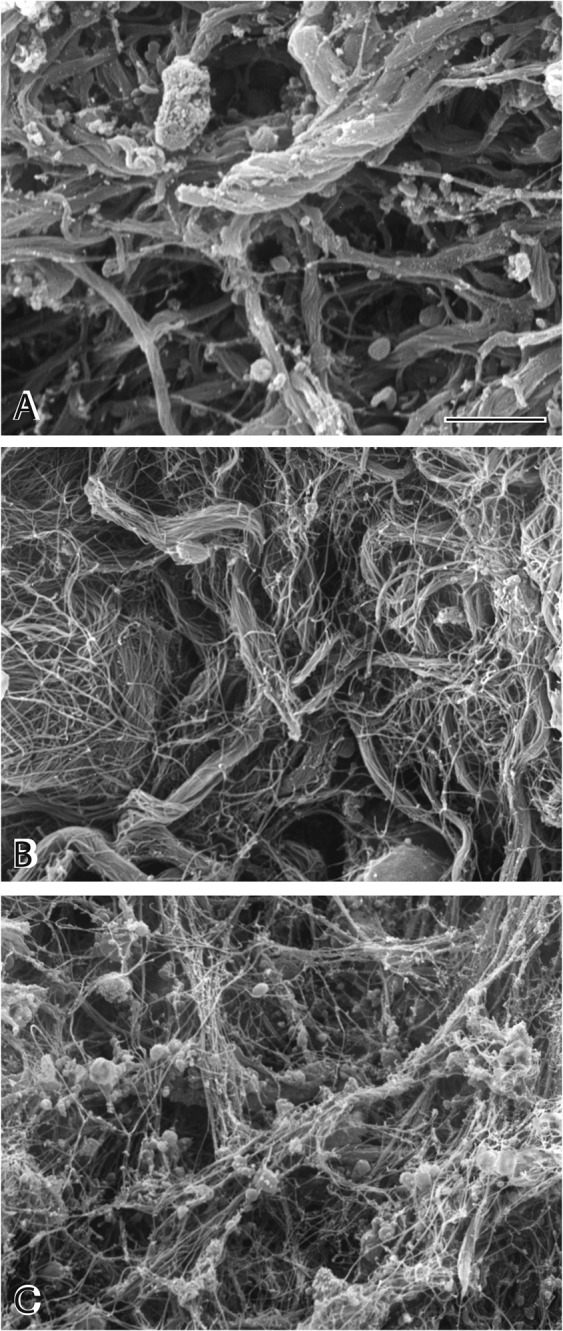
Fibrin structures in thrombi. There was great diversity in the structure of fibrin in thrombi and emboli. (**A**) Very thick bundles of fibers that are sometimes twisted with large pores. (**B**) Thick bundles of fibers made up of thinner fibers and large pores. (**C**) Thrombus with a non-uniform distribution of thin fibers with large pores and platelet aggregates and fragments. Magnification bar = 1 µm.

Three morphologically different fibrin structures were identified within all thrombi: individual fibrin fibers, fibrin bundles, and fibrin sponge. Fibrin fibers, which typically predominate in *in vitro* blood clots, are generally long and relatively straight with occasional branch points (Fig. 1E,F,I). The fibrin sponge is a fibrin network composed of very fine fibers, often with bound platelets and microvesicles (Fig. 1B). Fibrin bundles are fibers that have associated laterally with other fibers (Fig. 1A,D,G). Fiber bundles more than 1 μm in diameter were frequently observed within *ex vivo* thrombi (Fig. 3A,B), with very large pores separating bundles from each other (Fig. 3A). Some fibrin fibers formed twisted structures, especially thicker fibers or bundles (Fig. 3A). In some images, fiber bundles were present together with thin fibers and cells or cellular microvesicles (Fig. 3B,C). Fibrin fibers had either smooth or rough surfaces. In some sections, e.g. regions with many fiber ends, whole pieces of fibers appeared to be missing, perhaps as the result of partial enzymatic digestion^25^.

**Figure 4:**
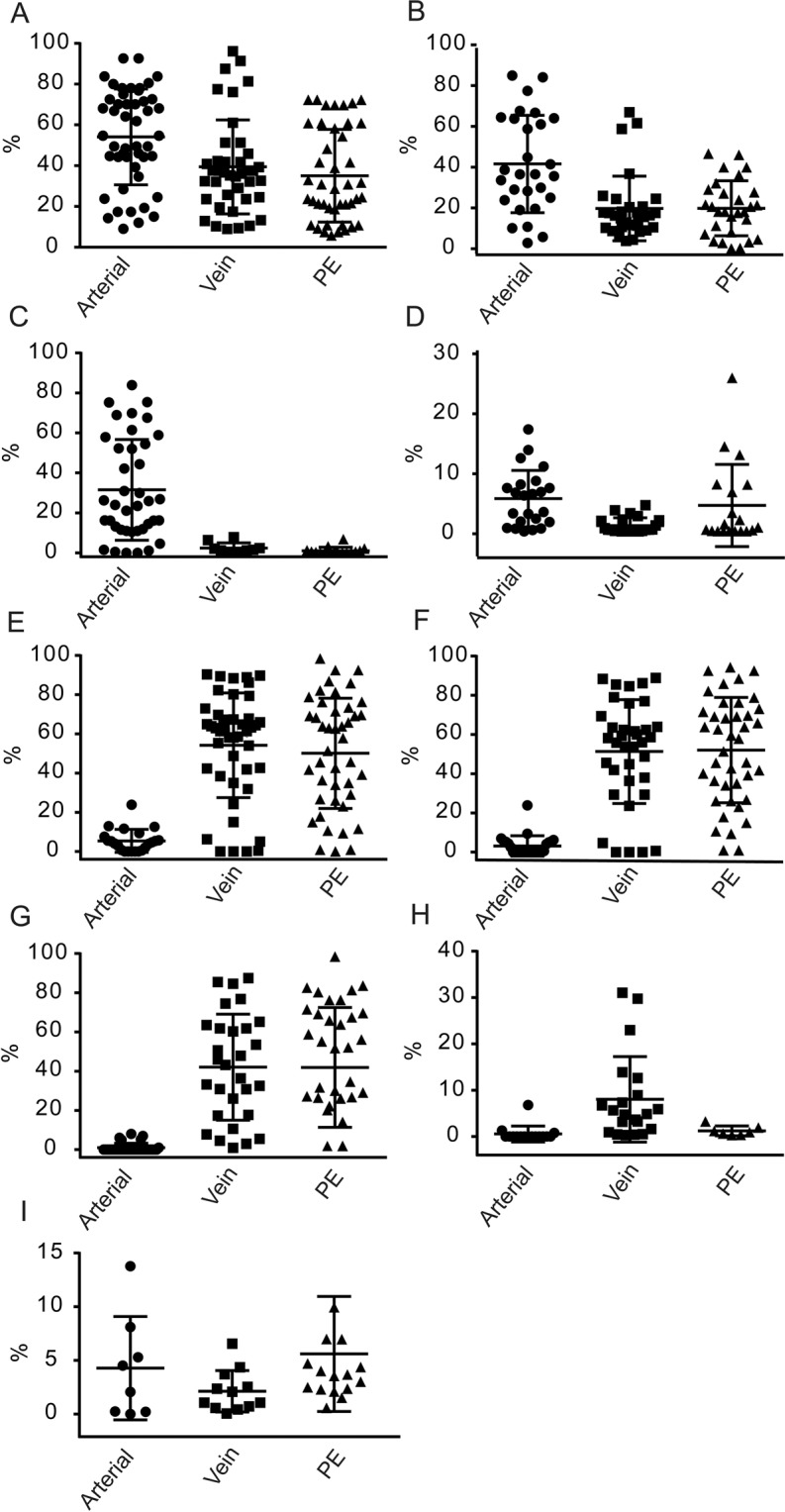
Quantitative analyses of structures (volume fraction, %) identified in pulmonary emboli, arterial and venous thrombi. Thrombi were visualized by scanning electron microscopy and their composition was quantified as described in the Materials and Methods section. The fibrin components, different shapes of erythrocytes as well as white blood cells, platelets and microvesicles were identified in thrombi and compared here as a percentage of the total thrombus volume. (•) – Arterial thrombi; (■) – Venous thrombi; (▲) – Pulmonary emboli. Quantitative differences were compared statistically by ANOVA test with Dunnett’s correction for multiple comparison. *P* values for differences between pulmonary emboli, venous and arterial thrombi are indicated by **, and *. (**A**) Fibrin fibers; **P = 0.0088, *P = 0.0408. (**B**) Fibrin bundles; **P = 0.0035, *P = 0.035. (**C**) Polyhedrocyte-shaped erythrocytes; **P < 0.0001, *P < 0.0001; (**D**) Platelets; **P < 0.001, *P = 0.002; (**E**) Microvesicles; **P = 0.048, *P = 0.010; (**F**) Echinocytes; **P = 0.0260, *P = 0.0068; (**G**) Total red blood cells; **P < 0.0001, *P < 0.0001; (**H**) Combination of polyhedrocytes and intermediate shapes of erythrocytes; **P < 0.0001, *P < 0.0001.; (**I**) White blood cells; **P = 0.0044, *P = 0.0034.

The average total fibrin content was greater in arterial thrombi (51 ± 5%) than in venous thrombi (36 ± 4%) or pulmonary emboli (31 ± 6%) (p = 0.0408 and p = 0.0088 respectively (Fig. 4A). More fibrin bundles were observed in arterial thrombi (42 ± 8%) than in venous thrombi (20 ± 3%) and pulmonary emboli (19 ± 8%) (p = 0.0035 for both (Fig. 4B).

**Figure 5:**
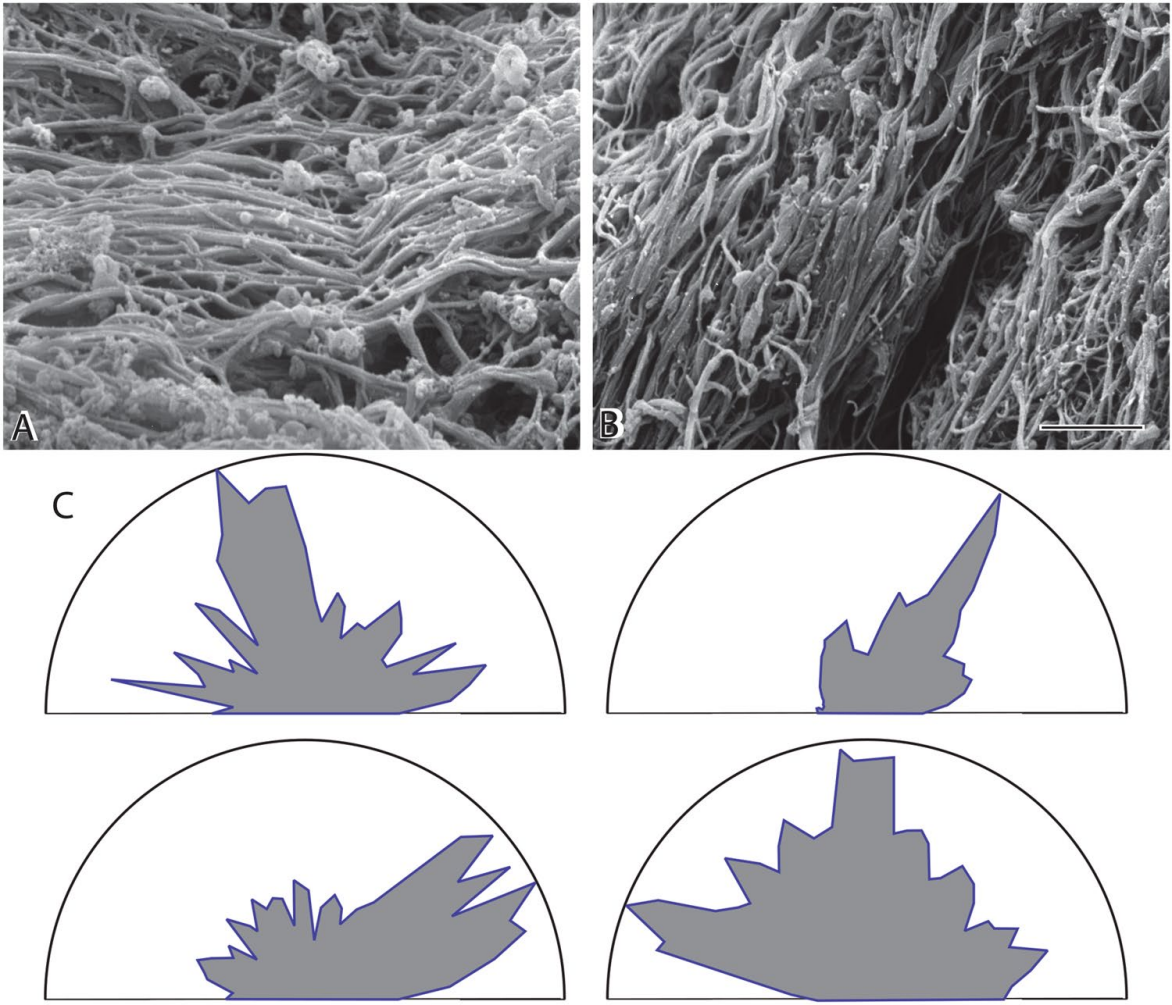
Orientation of fibrin fibers in thrombi. Some areas of some thrombi contained fibrin fibers largely oriented in a single direction. (**A**) Dense network of roughly horizontally oriented fibers. (**B**) Dense fibers oriented diagonally. (**C**) Polar plots of the frequency of fibers with different orientation. Distance ‘r’ represents the frequency of fibers with orientation at each angle between 0° and 180°, normalized to the maximum frequency. The angles are between 0°–180° rather than 0°–360°, because there is no directionality of the orientation. Each plot is from a single micrograph and the absolute value of the angle is arbitrary, but only relative to the other fibers in an individual image, which is why the values are not given on the coordinate for angles. Magnification bar = 1 µm.

In contrast to fibrin clots formed under static conditions *in vitro*, fibrin in thrombi showed the effects of flow. In thrombi, the fibers displayed a definite directionality or preferential orientation of fibers in a single direction, but the degree of anisotropy was highly variable. In some cases, parts of a thrombus with a dense mat of fibers were oriented in the same direction, with very few holes or gaps (Fig. 5A,B). The degree of anisotropy was quantified by the use of polar plots, showing numbers of fibers at different angles (Fig. 5C). Some areas contained fibers that showed relatively random orientation, while others showed a moderate or a large amount of alignment. In a few cases where the orientation could be determined from the gross structure of the thrombus, it could be seen that the fibers were oriented in the direction of flow.

Thrombi also showed effects of platelet-driven contraction on fibrin redistribution from being uniform throughout the clot to the surface (Fig. 3)^17^.

#### RBC content and morphology

The population of RBCs in the thrombi was divided morphologically into biconcave RBCs, intermediate-shaped RBCs, polyhedrocytes, balloon-like RBCs, and echinocytes. Polyhedrocytes originate from intravital platelet-driven mechanical compaction of thrombi. Intermediate-shaped RBCs are partially compressed cells that are no longer biconcave but not yet polyhedral-shaped. The total average RBC content was significantly greater in venous thrombi (50 ± 5%) and in pulmonary emboli (44 ± 6%) compared to arterial thrombi (3.7 ± 1.0%) (p < 0.0001 for both) (Fig. 4E). Interestingly, 45 ± 5% of RBCs in venous thrombi and 44 ± 6% in pulmonary emboli were present in intermediate and polyhedral-shaped forms, in contrast to only 3 ± 1% in arterial thrombi (p < 0.0001 for both). Surprisingly, venous thrombi had the greatest content of echinocytes (8 ± 2%) as compared to arterial thrombi (0.5 ± 0.1%) and pulmonary emboli (1.3 ± 0.2%) (p = 0.0260 and p = 0.0068 respectively) (Fig. 4H).

#### Platelet and extracellular microvesicle content

Platelets were a major component of arterial thrombi (35 ± 6%), but not of venous thrombi (2.3 ± 0.3%) or pulmonary emboli (1.0 ± 0.2%, p < 0.0001 for both) (Fig. 4C). All platelets found in arterial and venous thrombi and in pulmonary emboli were activated to different degrees with accompanying shape changes, most of them present as aggregates bound to fibrin.

All thrombi also contained clearly identified microvesicles, which may have derived from platelets, leukocytes, endothelial cells, or RBCs^26,27^. Microvesicle content was somewhat greater in arterial thrombi (5.0 ± 0.9%) and pulmonary emboli (3.5 ± 1.4%) than it was in venous thrombi (0.8 ± 0.2%) (p = 0.048, p = 0.010) (Table 1; Fig. 4D). There was a strong positive correlation between the content of platelets and microvesicles in both arterial and venous thrombi (r = 0.66, p = 0.022, and r = 0.87, p = 0.017, respectively) and a strong negative correlation between the content of microvesicles and the total fibrin component (r = −0.70, p = 0.05, for both arterial and venous thrombi). In pulmonary emboli, the correlation between microvesicles and platelets and microvesicles and total fibrin content was weak (r = 0.19, p = 0.033, and r = 0.22, p = 0.055, respectively).

#### WBC content

WBCs, identified based on their size being larger that RBCs and their cell surface projections (Fig. 1H), occupied only a small volume in arterial and venous thrombi and pulmonary emboli. However, the content of WBCs was significantly greater in pulmonary emboli (6.6 ± 3.3%) than in arterial (3.0 ± 1.2%) and venous thrombi (0.5 ± 0.2%) (p = 0.044 and p = 0.034, respectively) (Table 1; Fig. 4I).

#### Case reports

Location- and time-dependent changes in composition of arterial and venous thrombi can be gleaned from analyses of individual thrombi in the Case Reports section (see Supplement for details).

To answer the question whether composition and distribution of the various thrombus components depend on location and time of formation, we compared the head of venous thrombi, representing the oldest part attached to the vessel wall, with the tail, the most recently formed segment that extends in the direction of blood flow and with the middle (body) section lying between the two (Supplemental Figs. S2 and S3). The head and tail contained more fibrin than the body, while the body of the thrombus contained more RBCs than either the head or tail. There was a higher content of fibrin bundles in the head than in the tail. Polyhedrocytes predominated in the body of the thrombus compared with the head and the tail, where there were more intermediate-shaped RBCs.

Regional differences within arterial thrombi were studied in thrombi removed surgically from abdominal aneurysms. Those thrombi were large and were composed of many layers, much like an onion, that were separated by dissection (Supplemental Fig. S4). The more superficial (newer) layers were composed of a network of fiber bundles having fairly uniform diameters, i.e. few having large or small diameters, and regular spacing of pores, while fibrin fibers in the older (abluminal) layers of thrombus showed a much broader distribution of diameters ranging from very thin fibers to very thick fiber bundles, often with a rough surface. Similar results were seen upon examination of two arterial graft thrombi from the same patient, one only a few hours old and the other 2 days old.

## Discussion

In the present study, the composition of arterial and venous thrombi and pulmonary emboli was characterized quantitatively using high-resolution scanning electron microscopy. Analysis and quantification of these images is extremely laborious and time consuming, since it must be done manually, taking about 3–4 hours per image for an experienced scientist. Consequently, not all images could be quantified, but the conclusions were entirely consistent with qualitative results from examination of images from all thrombi and emboli. The complexity of the study dictated several unavoidable limitations, such as the inter-observer disagreement of the order of 5–10% and the possibility of postmortem changes in pulmonary emboli.

Although traditionally arterial thrombi have been termed “white” and venous thrombi “red”, additional detail about the similarities or difference in their composition has been limited. We extended that knowledge to the quantitative level and show that arterial thrombi contain a surprisingly large amount of fibrin, even more than the volume occupied by platelets.

Our results confirm prior studies showing that the proportion of RBCs in venous thrombi and pulmonary emboli is considerably higher than in arterial thrombi. Compressed polyhedral RBCs, termed polyhedrocytes, and intermediate-shaped forms (both indicative of the intravital compaction of a thrombus mass), were also more common in venous thrombi and pulmonary emboli than in arterial thrombi. More fibrin bundles were observed in arterial than in venous thrombi and pulmonary emboli, but more individual fibrin fibers were observed in pulmonary emboli than in arterial and venous thrombi. WBCs were found more commonly in pulmonary emboli compared with arterial and venous thrombi, while microvesicles were more common in arterial thrombi and pulmonary emboli.

These differences in composition may arise from the conditions under which the thrombi formed, as follows. Thrombus structure is affected by constriction of the vessel wall, activation or damage of the endothelium, blood flow and shear, fibrin polymerization, platelet-platelet and platelet-fibrin interactions, and the effect of platelet contractile forces on fibrin fibers and cells. Arterial thrombi form under high shear rates of 500–1500 s^−1^, which significantly increases with stenosis as the thrombus grows, and may reach up to 250,000 s^−1^ ^28,29^. Increasing shear rate has a large impact on the fibrin network structure, promoting orientation and association of fibrin fibers into bundles^30–34^. We quantified this orientation in some thrombi, which is in agreement with our finding that arterial thrombi assembled at high shear rate contain more thick bundles (some twisted) than venous thrombi assembled at low shear rates. Arterial thrombi had higher platelet content, in agreement with earlier histological results^35^, which may contribute to fibrin bundle formation, since platelets pull on fibrin fibers^36^, decreasing the space between them, thereby promoting lateral association of fibers into bundles^37^. Another mechanistic aspect of these results is that the main structural outcomes of clot contraction are the redistribution of the platelets and fibrin toward the periphery of the clot and compression of erythrocytes in the interior of the clot, followed by their deformation into polyhedral cells that we have named “polyhedrocytes”. These structural signatures of clot contraction in *ex vivo* thrombi and thrombotic emboli derived from different vessels in human patients demonstrate that intravital contraction is common *in vivo*^17^.

Our results also confirm and extend previous studies by demonstrating that RBCs are found within arterial thrombi. The relative paucity of RBCs in arterial compared with venous thrombi can be attributed primarily to the high shear rate, but it is notable that a large fraction of those that were present were polyhedrocytes. In addition, many balloon-shaped RBCs were evident (Fig. 1C), perhaps indicative of RBCs partially escaping from a contracting arterial thrombus.

Polyhedrocytes that form during clot contraction were discovered initially in clots made *in vitro*, but the present studies extend this observation by showing their presence in all clinical thrombi examined. Indeed, compressed RBCs comprised the major part of human venous thrombi and pulmonary emboli, demonstrating that they are not simply *in vitro* artifacts. Moreover, the prevalence of polyhedrocytes in thrombi is a morphological signature of clot contraction that is quite widespread in thrombi *in vivo*. Not all RBCs assumed a polyhedral shape, since we observed intermediate forms and balloon-like shapes as well. These differences likely reflect a balance between the strength of platelet-derived contractile forces and the cellular composition of the clot that resists contraction^21^. Although venous thrombi and pulmonary emboli contain fewer platelets than arterial thrombi, the proportion of RBCs that were polyhedrocytes was even greater than in arterial thrombi. This seeming paradox might be explained by the recently described disintegration of thrombin-activated platelets after clot formation and contraction^19,38^ and perhaps the effect of muscle pumps from the surrounding skeletal muscles that can develop compression stresses up to 0.5 MPa and may contribute to the compaction of thrombi^39^.

Surprisingly, the amount of space between structures was quite small, less than 2%, in both thrombi and emboli. A likely major consequence of this very small amount of space between structures is that permeability of pathological clots would be extremely low, much lower than would be estimated based on studies of *in vitro* clots, which may limit access of therapeutic agents and account for the limited effectiveness of thrombolysis.

Unexpectedly, venous thrombi, especially the head (oldest part), had the greatest content of echinocytes as compared to arterial thrombi and pulmonary emboli. This finding is in agreement with some evidence that the transformation of RBCs into echinocytes is related to an increase in blood viscosity^40,41^ that may occur due to turbulence, stasis, as well as the resulting hypoxia or metabolic exhaustion of RBCs. Many fewer echinocytes were observed in pulmonary emboli and arterial thrombi, suggesting that the difference in the environment of established venous thrombi leads to a transformation to echinocytes in favor of other forms of RBCs.

The composition of pulmonary emboli was distinctly different from their presumptive origin in the venous system. Pulmonary emboli contained a higher proportion of fibrin than venous thrombi, especially of individual fibers, and fewer total RBCs, especially biconcave and intermediate forms and a greater content of WBCs and microvesicles (Fig. 2*)*, It is not yet clear how these differences in composition might be related to the risk of embolization, or to changes after embolization *in vivo*. Because the emboli were obtained from cadavers, some postmortem alterations could potentially affect their composition, but fresh samples could not be obtained and these samples were processed as soon after death as possible; moreover, the lack of morphological signs of fibrinolysis^25^ reassures us that the impact of postmortem changes was limited.

Some support for the biological significance of these observations comes from the similarities between the composition of the tail of a venous thrombus from which embolization is most likely to originate, compared with those found in the body and head of the same thrombus (Supplemental Fig. S3D,I), although a limitation of this aspect of the study is that only one such thrombus was available. The tail region of this venous thrombus, like the emboli, contained more fibrin and fewer RBCs than the other segments. These results are consistent with experiments in an animal model showing that the composition of venous thrombi change over time^42^. Initially RBCs were the predominant constituent of thrombi, but later fibrin increased in the head and tail, while the body was composed primarily of RBCs, in striking agreement with our findings. The bodies of venous thrombi were composed mostly of polyhedrocytes, whereas intermediate-shaped RBCs predominated elsewhere. The higher content of fibrin fibers in the head and tail may impede the platelet contractile forces, reducing formation of polyhedrocytes. This may impair contractility, making this region more prone to embolization, because impaired clot contraction has been shown to correlate with a higher incidence of pulmonary embolism in patients with deep venous thromboembolism^43^.

There appear to be similarities and differences in the aging of arterial thrombi compared with venous thrombi. Two independent approaches were taken to examine changes that occur in the fibrin of arterial thrombi as they age. Thrombi associated with aortic aneurysms are composed of layers that can be separated from each other and likely represent different stages (and ages) in clot development. In addition, in the one case available for review, a newly formed thrombus was removed at the same time as an older thrombus, so that their structures could be compared. The conclusions from both of these approaches were similar. Older arterial thrombi were denser, containing many more fibrin fibers with fewer intervening pores. This increase in density may reduce permeability through the thrombus leading to increasing resistance to fibrinolytic agents over time.

Platelets present in older thrombi were sometimes perforated by holes (Supplemental Fig. S4E) that likely arise from degranulation^44^, but most changes were observed in the structure of fibrin. In arterial thrombi, the fiber bundles in older thrombi were much less homogeneous in diameter. Older thrombi were made up of both more fibers that are thicker and more fibers that are thinner, consistent with addition of fibers over time, thickening preexisting fibers, while those forming in the interstices would appear as thin fibers. Older portions of thrombi also displayed thick fiber bundles with rough surfaces, in contrast to the smooth surfaces of fibers in newer thrombi. The rough surfaces of fibers in older portions of a thrombus may arise from both adherent microvesicles and clusters of proteins that are known to bind to fibrin, such as plasminogen, tissue-type plasminogen activator, plasminogen activator inhibitor-1, α2-antiplasmin, fibronectin, as well as DNA^45–47^.

## Conclusions

We have characterized in detail the structure and composition of human arterial and venous thrombi and pulmonary emboli. We showed reproducible distinctive differences among all three related to their origin and destination. Although some general results, such as arterial thrombi having the gross appearance of being “white” and venous thrombi being “red”, have been known for many years, there were some surprises, such as the preponderance of fibrin in all of these structures, providing a rationale for thrombolytic therapy for both arterial and venous thrombi. Moreover, all types contained substantial numbers of RBCs, most of which were compressed by platelet-driven clot contraction into polyhedrocytes and intermediate-shaped forms, with very few uncompressed biconcave cells. Some echinocytes were present in venous thrombi, perhaps as a result of exhaustion of RBC metabolic functions over time. There was very little space between fibrin structures in all thrombi and emboli, far less than expected based on *in vitro* findings, which likely reduces permeability *in vivo* and explains the limited efficacy of thrombolytic therapy. There were differences in fibrin and RBC content between pulmonary emboli and deep venous thrombi, similar to differences in the older head, middle, and younger tail of venous thrombi, which might relate to causes of embolization. Distinctive morphological differences were observed in fibrin structures of different age, which can be accounted for by increasing accumulation of fibrin over time. These results may have important clinical implications for the understanding and treatment of thrombotic disorders.

## Material and Methods

### Clinical material and ethical aspects

Arterial and venous thrombi and pulmonary emboli were obtained from patients receiving standard antithrombotic treatment with no differences in terms of gender or age. The ethical aspects are described in detail in the Supplement, the “Clinical material and ethical aspects” section. The study was approved by Institutional Review Board (IRB) of University of Pennsylvania and local IRBs and Ethical Committees of the Institution that participated in the study. All methods were performed in accordance with the relevant guidelines and regulations, including informed written consent obtained from all patients.

Coronary arterial thrombi from patients with ST-elevation myocardial infarction (n = 45), venous thrombi (n = 25) and pulmonary emboli (n = 10) were obtained as described in detail in the Supplement, the “Clinical material and ethical aspects” section. No morphological signs of fibrinolysis such as fibrin fibers ends and rifeness of fibers^25^ were observed in any intravitatal or postmortem samples.

### Scanning electron microscopy

All thrombi and thrombotic emboli were prepared as described in detail in the Supplement and processed as described previously^48^. High-definition micrographs were obtained from different randomly chosen areas of each thrombus or embolus to eliminate selection bias (Supplemental Fig. I) and examined in a Quanta 250FEG scanning electron microscope (FEI, Hillsboro, OR). The predominant structures within these samples were identified based on previous experience^8,17,24,38,49–51^, and representative images were assessed for all structures listed in detail in the Data Supplement.

### Image quantification and statistical analysis

Quantitative assessment of thrombi and thrombotic emboli composition was carried out using the same previously used procedures^7,24^, which are described in detail the Data Supplement. Briefly, election micrographs were analyzed for each specimen individually, using a grid placed over each micrograph (Supplemental Fig. I). Each structural component was quantified using an approach described in detail in six steps in the Data Supplement, to avoid any artifacts from any source. The significance of the differences in composition of the three types of samples (arterial thrombi, venous thrombi, and emboli) was analyzed using the Chi-square test with a 95% confidence level (p < 0.05). As an independent approach, the composition of thrombi and emboli was presented as mean values averaged over all quantified electron micrographs obtained for all 5 or 6 samples of thrombi/emboli. The results of the relative volume fractions were expressed as a mean ± standard error of the mean after testing for normality using the Kolmogorov-Smirnov criterion. Those data were compared statistically by a one-way ANOVA test with Dunnett’s correction for multiple comparisons with a 95% confidence level (p < 0.05). Associations between contents of various structural components were assessed by the Pearson’s correlation test if the data arrays met the assumption of normality. All statistical analyses were carried out with GraphPad Prism version 6.07 (GraphPad Software, Inc., La Jolla, CA, USA).

See details for the statistical analyses in the Data Supplement.

## Supplementary information

Supplementary Material .
