# Supplementary material for: The distinctive structure and composition of arterial and venous thrombi and pulmonary emboli
Source: Sci Rep. 2020 Mar 20;10:5112. doi: 10.1038/s41598-020-59526-x (PMC7083848; doi:10.1038/s41598-020-59526-x)
Supplement: Supplementary file 1 — Supplementary Material . [file 41598_2020_59526_MOESM1_ESM.docx]

**Supplementary Material**

**The distinctive structure and composition of arterial and venous thrombi and pulmonary emboli**

Irina N. Chernysh^1^, Chandrasekaran Nagaswami^1^, Sofia Kosolapova^1^, Alina D. Peshkova^2^, Adam Cuker^1^, Douglas B. Cines^1^, Carolyn L. Cambor^1^, Rustem I. Litvinov^1,2^,

and John W. Weisel^1^

^1^University of Pennsylvania School of Medicine, Philadelphia, PA, USA, ^2^Kazan Federal University, Kazan, Russian Federation

**Material and Methods**

**Clinical material and ethical aspects**

Arterial and venous thrombi and pulmonary emboli were obtained from patients receiving standard antithrombotic treatment with no significant differences in terms of gender or age. The study was approved by Institutional Review Board (IRB) of University of Pennsylvania and local IRBs and Ethical Committees of the Institution that participated in the study. All methods were performed in accordance with the relevant guidelines and regulations, including informed written consent obtained from all patients.

Intracoronary arterial thrombi were obtained during aspirational thrombectomy from 45 patients with ST-elevation myocardial infarction as part of clinically indicated percutaneous interventions performed at the Institute de Cardiology of Pitié-Salpêtrière Hospital, University of Paris, France. The procedure was performed for patients with a Thrombolysis In Myocardial Infarction (TIMI) flow grade 0 or a visible thrombus if TIMI flow grade was scored at 1 or higher, demonstrated by coronary angiography. Thrombi were aspirated using a low-profile catheter (Export 6F, Medtronic, Santa Rosa, California) whenever the size and curvature of the coronary artery permitted. Major clinical characteristics of the patients with coronary arterial thrombosis included in the study are provided in Table S1.

**Table S1. Clinical characteristics of patients with coronary arterial thrombosis included in this study**

| *Patients with arterial (coronary) thrombosis (n=45)* | |  |
| --- | --- | --- |
| Age (years) 57.9 ± 13.2 | | |
| Gender | Men 38 (84.4%) | |
|  | Women 7 (15.6%) | |
| Fibrinogen level | 3.7 ± 1.3 g/L | |
| Infarct-related artery | left anterior descending 21 (46.6%) | |
|  | right coronary artery 17 (37.7%) | |
|  | circumflex 6 (13.3%) | |
| TIMI score | 0-3 18 (40%) | |
|  | 4-7 27 (60%) | |
| Abciximab | No 4 (8.9%) | |
|  | Yes 41 (91.1%) | |
| Thrombolysis | No 44 (97.8%) | |
|  | Yes 1 (2.2%) | |
| Thrombus age (hrs) | 4.1 ± 0.3 | |
| Heparin | Enoxaparin 26 (57.8%) | |
|  | Unfractionated 17 (37.8%) | |
| Aspirin | No 0 (0%) | |
|  | Yes 45 (100%) | |
| Clopidogrel | No 8 (17.8%) | |
|  | Yes 37 (82.2%) | |
| Major bleeding | No 43 (95.6%) | |
|  | Yes 2 (4.4%) | |
| Minor bleeding | No 35 (77.8%) | |
|  | Yes 10 (22.2%) | |
| Death in the hospital | No 44 (97.8%) | |
|  | Yes 1 (2.2%) | |
| Death at day 30 | No 42 (93.3%) | |
|  | Yes 3 (6.7%) | |

Venous thrombi (n=25) were obtained by open thrombectomy in the femoral-popliteal or iliofemoral segments of the common femoral vein. The entire floating part of the thrombus (from the place of attachment to the vessel wall to the apex of the intraluminal segment was removed from the opened vein with a terminal clamp or forceps with minimal mechanical perturbation, rinsed with a physiological buffer and placed in a tube with 2% glutaraldehyde as a fixative. The study of venous thrombi was approved by the Ethical Committee of the Interregional Clinical Diagnostic Center (ICDC, Kazan, Russia) protocol No. 70 on January 30, 2016, and informed written consent was obtained from all patients. Based on the site of thrombotic occlusion, the samples were designated as distal thrombosis of the femoral-popliteal segment (8 or 32% of the patients) or proximal iliofemoral thrombosis (17 or 68% of the patients). Based on the duration of symptoms, samples were designated as acute (<21 days) or subacute (>21 days) thrombosis. Patients with suspected PE underwent a CT scan of the chest with contrast that confirmed the diagnosis. Major clinical characteristics of the patients with venous thromboembolism included in the study are provided in Table S2.

**Table S2. Clinical characteristics of patients with VTE included in this study**

| ***Patients with venous thrombosis*** *(n=25)* | |  |
| --- | --- | --- |
| Age (years) | | 60.8 ± 8.5 |
| Gender | Men | 16 (64%) |
|  | Women | 9 (36%) |
| Floating part of the thrombus | >7 cm | 3 (12%) |
|  | <7 сm | 22 (88%) |
| Location of the thrombus | Proximal | 17 (68%) |
|  | Distal | 8 (32%) |
| Duration of symptoms | >21 days (subacute) | 3 (12%) |
|  | < 21 days (acute) | 22 (88%) |
| Pulmonary  embolism | No | 10 (40%) |
|  | Yes | 15 (60%) |

Mural thrombi from abdominal aortic aneurysms (n=3) were obtained from patients undergoing vascular surgery at the Hospital of the University of Pennsylvania (Philadelphia, Pennsylvania, USA) with informed written consent.

Pulmonary emboli (n=10) were obtained during autopsies performed in the Department of Pathology, Hospital of the University of Pennsylvania (Philadelphia, Pennsylvania, USA). These specimens from pulmonary arteries were all large (several centimeters) and from patient review most likely arose from the lower limbs. Changes that might have occurred post mortem are unknown but no morphological signs of fibrinolysis (such as cut fibers or rough fiber surfaces) or other post-mortem changes were observed. Major clinical characteristics of the 10 deceased patients with pulmonary embolism (PE) included in the study are provided in Table S3.

**Table S3. Clinical characteristics of patients with PE included in this study**

| *Patients with PE (n=10)* | |  |
| --- | --- | --- |
| Age (years) | | 58 ± 14 |
| Gender | Men 5 (50%)  Women 5 (50%) | |
| Prior history of DVT or PE |  | 2 (20%) |
| Comorbidities | Hypertension 7 (70%)  Diabetes 2 (20%) | |
| Risk factors | Cancer (lung, brain) 1 (10%)  Postoperative state 2 (20%)  Immobility or decreased mobility 5 (50%)  Obesity 2 (20%) | |
| Postmortem interval | ~20-30 h | |
| Cause of death | Directly due to PE/saddle embolus 5 (50%)  PE contributed to multifactorial causes of death 5 (50%) | |
| Location of PE | Saddle PE 1 (10%)  Right or left main pulmonary arteries 5 (50%)  Lobar pulmonary artery 2 (20%)  Segmental and subsegmental pulmonary artery 2 (20%) | |

**Scanning electron microscopy**

All thrombi and thrombotic emboli were placed in saline solution immediately after their removal. Specimens were rinsed with saline and fixed in 2% glutaraldehyde in 50 mM sodium cacodylate buffer with 150 mM NaCl (pH 7.4) and processed as described. Some thrombi were examined intact, while larger pieces were dissected to compare the interior and exterior segments. High-definition micrographs were obtained from 10-12 different randomly chosen areas of each thrombus or embolus to eliminate selection bias (Supplemental Figure 1) and examined in a FEI Quanta 250FEG scanning electron microscope (FEI, Hillsboro, OR). The predominant structures within these samples were identified based on previous experience^1, 4^ and representative images were assessed as described below.

The following structural elements were identified in the images by experienced observers. Fibrin was present in three distinctive forms: (1) individual fibers making up a branched network as in plasma clots, (2) bundles of fibers, as seen previously in mechanically deformed clots, and (3) a newly discovered form of fibrin consisting of a highly branched network of very thin fibers, which we named “fibrin sponge.” Platelets were present either as individual cells, most of which appeared to be highly activated from shape changes, as aggregates, or as degranulated platelet remnants identified by the presence of apparent “holes” in the membrane. Red blood cells (RBCs) were present in several forms, i.e. as typical biconcave cells, as echinocytes (identified by spikey protrusions), or as one of three forms compressed to a variable degree as has been identified previously within contracted clots: polyhedral RBCs (“polyhedrocytes”), forms intermediate between biconcave and polyhedral, and balloon-like cells. White blood cells were identified based on their larger size and surface features known from the literature. Cellular microvesicles were characterized as round structures smaller than 1µm. Space between structures was also quantified.

**Image quantification and analysis**

All thrombi and emboli were examined by scanning electron microscopy, and many images were collected. Qualitative conclusions were established by examination of all images by at least 3 independent observers who were blinded to the source of the material except the lead organizer. Internal reproducibility was established by re-quantifying a representative set of images from some of the same specimens by different examiners. After observers were trained, inter-observer variation was negligible. Patterns in the structure and composition were visually apparent by observations of hundreds of images of each group made simultaneously. Not all images could be quantified because this process takes many hours per image. Therefore, a subset of 5 venous thrombi, 6 arterial thrombi, and 6 pulmonary emboli were randomly selected for more in depth quantitative analysis provided that technically high quality images could be obtained. Quantitative assessment of thrombi and thrombotic emboli composition was carried out using previously described procedures.The specimens were sectioned and 10-12 micrographs of each from randomly selected areas were taken at 2,000× magnification. The images were transposed onto a computer screen and a fine grid (1.5 μm × 1.5 μm) was overlaid using Image J 1.48 software (National Institutes of Health, Bethesda, MD, USA) (Supplemental Figure 1). Each image was divided into these squares such that usually only one predefined structural element, as defined above, was present in each. If the square was occupied by two structures in the same ratio, each would be counted as ½ structure, or similarly for other types of multiple occupancy.

The total numbers of each type of structure in each micrograph were thus counted manually. Deeper structures in each image were disregarded. Because the micrographs provide representative images from all portions of the thrombi and thrombotic emboli, including the interior and exterior, analysis of many individual images can provide a picture of the entirety of the structural composition of the specimen, and hence the volume occupied by each type of structure can be estimated. The basic principle is that multiple multidirectional two-dimensional views or sections of the interior and exterior of an object give three-dimensional information. In this case, multiple views of many specimens were quantified to yield information not about a single object but rather the overall structure of all specimens in each class.

**Statistical Analyses**

To carry out an accurate quantitative determination of the composition of the selected specimens, 10-12 election micrographs were analyzed for each specimen individually, using a grid placed over each micrograph as described above. Each structural component (as described above: 3 different fibrin structures, 5 different shapes of RBCs, white blood cells, platelets, microvesicles, and empty spaces between structures) was calculated by summarizing the area (as the number of 1.5μm x 1.5μm squares of the grid) occupied by each particular structural component in every electron micrograph (see Methods and Supplemental Figure 1). To represent each structural component of all arterial or venous thrombi or emboli as a whole (in pie charts and tables) and minimize any artifacts from differences in size of the samples or numbers of images, the following approach was used:

1. The numbers of grid squares (in 1.5μm x 1.5μm) occupied by each structural component, or partial grid squares if more than one structure was present, obtained from individual micrographs were summarized for all micrographs pertaining to one sample, either thrombus or embolus.

2. The resulting number of grid squares occupied by each structural component within one sample (thrombus or embolus) were summarized separately and then the sum of squares of each structural component was divided by the total number of grid squares analyzed for all structural components of the sample, thus providing the fraction of area occupied by each structural component within one sample (thrombus or embolus).

3. The number of grid squares occupied by each structural component in each sample was summarized for all 5 or 6 samples of a certain type.

4. The number of grid squares of all structural components for each sample was divided by the total number of grid squares of all structural components measured for all 5 or 6 samples of a certain type (thrombus or embolus), providing the fraction of each sample (thrombus or embolus) out of all 5 or 6 samples (for that category of thrombi or emboli).

5. The fraction of relative area occupied by each structural component within one sample (calculated in #2 above) was multiplied by the fraction of area of this sample in all samples (calculated in #4 above). Thus, each structural component was evaluated with respect to each sample as well as with respect to all samples.

6. The final corrected relative areas (corresponding to volume fractions) occupied by each structural component in each type of thrombi/emboli were summarized and presented as pie charts. Thus, the final numbers were corrected (or weighted) for the area analyzed for each sample because the area that was analyzed varied from sample to sample.

**Case Reports**

*Location and time dependent differences within venous thrombi*

We observed differences in the distribution of the two major components of the thrombus, fibrin and RBCs, in the head, body and tail of the same thrombus (Supplemental Figure S2). The head and tail contained more fibrin (each 34%) than the body (13%, p=0.03) (Supplemental Figure SA). The body of the thrombus contained more RBCs than either the head or tail (84% vs. 63% and 65%, respectively, with p=0.031 for both) (Supplemental Figure S3 A). The morphology of the fibrin differed, with a higher content of bundles in the head than in the tail (22 *±* 4% vs. 14 *±* 3%), (p=0.02) (Figure S3 C). The content of fibrin bundles in the body (11 *±* 1%) was similar to that in the tail (10 *±* 1%, (p=0.056). Polyhedrocytes predominated in the body of the thrombus (77 *±* 4%) compared with the head (20 *±* 8%) and the tail (35 *±* 12%) (p=0.001 and p=0.013 respectively) (Supplemental Figure S3 E). In contrast, intermediate-shaped RBCs predominated within the head and tail (29 *±* 10% and 31 *±* 8%, respectively), compared to the body (5 *±* 3%, p=0.031 and p=0.0173, respectively) (Supplemental Figure S3). Echinocytes were more prevalent in the head of the thrombi (20 *±* 5%) than in the tail (2.1 *±* 0.7%) or body (1.1 *±* 0.9%) (p=0.098, p=0.003 respectively) (Supplemental Figure S3 G). Microvesicles were somewhat more common in the head (3.5 *±* 0.7%) than in the body (1.5 *±* 0.2%) and tail (0.9 *±* 0.1%) (p=0.09 for both).

*Location and time differences within large arterial thrombi*

The composition of the deeper, i.e. older, layers with more superficial, i.e. newly formed layers were compared. There was a gradual, continuous transition from structures present in the deeper layers to those present at the luminal surface, resulting in distinct differences based on distance from the aneurysm wall. Quantification of the images was precluded by the small sample size (n=3) and sample-to-sample variability. However, remarkable qualitative differences between the layers were evident, especially in the fibrin structures. The more superficial (newer) layers were composed of a network of fiber bundles having fairly uniform diameters, i.e. few having large or small diameters, and regular spacing of pores (Supplemental Figure 4A). Platelet aggregates were quite common while RBCs were sparse. In contrast, fibrin fibers in the older (abluminal) layers of thrombus showed a much broader distribution of diameters ranging from very thick fiber bundles to some very thin fibers (Supplemental Figure S4D, E). The few RBCs that were apparent were in the form of polyhedrocytes. Many of the platelets tended to have holes in their plasma membrane, perhaps as a result of degranulation. The thick fiber bundles in images of more abluminal (older) layers of the thrombi often appeared to have a rough surface (Supplemental Figure S4D), while the fibers in the luminal (younger) surface were smoother (Supplemental Figure S4A), similar to the appearance of plasma clots. The rough appearance of fibers from abluminal layers appeared to arise from particles on their surface (Supplemental Figure S4E)*.*

Similar results were seen upon examination of two arterial graft thrombi from the same patient, one only a few hours old and the other 2 days old. Again, the more recent thrombus was composed of fibers of uniform diameter with large pores (Supplemental Figure S4B, C), whereas the older thrombus was made up of a much more heterogeneous collection of fibers (Supplemental Figure S4F). The fiber density was also much greater in the older thrombus and the fiber bundles often had a rough surface. Although these results must be interpreted cautiously since graft thrombi are different in origin than the other thrombi studied here, the newer thrombus was similar in appearance to the luminal surface of the thrombi from the patients with an aortic aneurysm, while the older thrombus was similar to the abluminal layers of the aortic aneurysm thrombi.

**Supplemental Figures**

**Figure S1. Analyses of structures and composition of thrombi and emboli.** (a) Low magnification of thrombus prepared for scanning electron microscopy. Black squares indicate areas that were used to quantify the thrombus structures. Magnification bar = 0.6 μm. (b) Scanning electron micrograph of a thrombus with a grid (1.5 μm × 1.5 μm each square) overlaid on the top of the image. The size of each grid square is about the size of the smallest structures, so that each square ordinarily contained only one type of structure, an entire structure or its part. Magnification bar = 10 µm. (c) A zoomed in area from the micrograph in B, as shown by the red box.


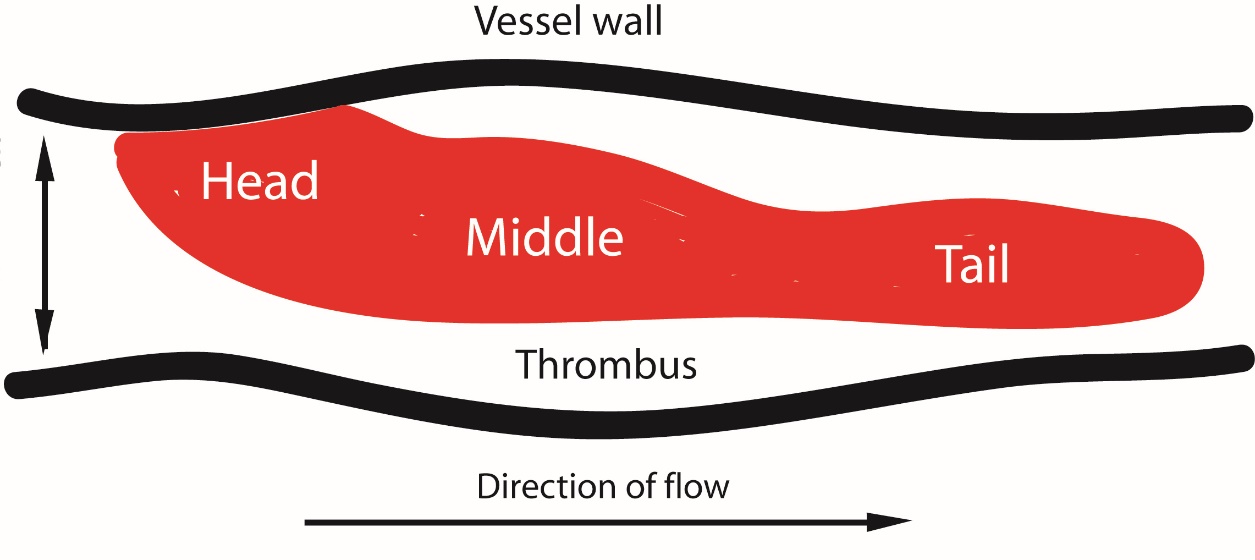


**Figure S2. Schematic diagram of parts of a deep vein thrombus.** The head of the thrombus is attached to the vessel wall, while the tail and middle portions are floating downstream. Embolization is most likely to occur from the tail of the thrombus.

**Figure S3. Quantitative analyses of structures identified in the head, middle and tail of a venous thrombus.** The head, middle and tail of the thrombus identified by the surgeon were dissected from venous thrombus and prepared for scanning electron microscopy and quantified as described in the Materials and Methods section. (a) The composition and heterogeneity of the three regions of this venous thrombus displayed as pie charts. RBC=red blood cell. (b – g) Bar charts showing the quantitative comparison of components of three parts of the thrombus. Black bars – head; White bars – middle; Grey bars – tail. The amounts of each component were compared statistically by ANOVA test with Dunnett’s correction for multiple comparison. *P* values for differences between head, middle and tail parts of the thrombus are indicated by **, and *. (b) Fibrin fibers; ***P*=0.0317, **P*=0.0330. (c) Fibrin bundles; **P*=0.002. (d) Red blood cells; **P*=0.046; (E) Polyhedrocytes; ***P*=0.001, **P*=0.0130; (f) Intermediate shapes of RBCs; ***P*=0.0317, **P*=0.0173; (g) Echinocytes; ***P*=0.0098, **P*=0.0047.

**Figure S4.** **Structure of older versus newer thrombi**. (a-c) Younger thrombi. (d-f) Older thrombi. (a)(d)(e) Layers of an aortic aneurysm. (b)(c)(f) Older and newer thrombi in the same patient. (a) Luminal layer of aortic aneurysm thrombus, representing newer layer. The fibrin network is typical of that in some whole blood clots, with a relatively uniform array of branching fibers, with biconcave red blood cells and a monocyte visible. (b) Newer thrombus in femoral-popliteal graft, removed by thrombectomy. A branching network of relatively uniform fibrin fibers is present with some platelet fragments and a few compressed red blood cells. (c) Another image of the newer thrombus in femoral-popliteal graft, removed by thrombectomy. A relatively uniform array of partially oriented fibrin fibers is present with a few platelet fragments. (d) Abluminal layer of aortic aneurysm thrombus, representing older layer. Fibrin fibers with widely different diameters are present and most are coated with many microvesicles. (e) Another image of abluminal layer of aortic aneurysm thrombus, representing older layer. Many platelets are present with holes from release of their granule contents. (f) Older thrombus in aorto-femoral bypass graft, removed by thrombectomy. Fibrin fibers are mainly quite thick with large pores. Magnification bar = 1 μm.
